# Absolute versus change in pulmonary vascular resistance in relation to European Society of Cardiology/European Respiratory Society risk change in pulmonary arterial hypertension

**DOI:** 10.1016/j.jhlto.2026.100599

**Published:** 2026-05-20

**Authors:** Stijn C.M. Donker, Christopher J.B. Wild, Daniel X. Augustine, Jay Suntharalingam, Robert V. MacKenzie Ross, Arie P.J. van Dijk, Joseph D. Maxwell, David Oxborough, Dick H.J. Thijssen

**Affiliations:** a Department of Medical BioSciences, Radboud University Medical Center, Nijmegen, the Netherlands; b Research Institute for Sport and Exercise Sciences, Liverpool John Moores University, Liverpool, United Kingdom; c Department of Cardiology, Royal United Hospitals Bath NHS Foundation Trust, Bath, United Kingdom; d Department for Health, University of Bath, Claverton Down, Bath, United Kingdom; e Department of Respiratory Medicine, Royal United Hospitals Bath NHS Foundation Trust, Bath, United Kingdom; f Department of Cardiology, Radboud University Medical Center, Nijmegen, the Netherlands

**Keywords:** Pulmonary arterial hypertension, pulmonary vascular resistance, right heart catheterization, treatment response, risk stratification, haemodynamic changes

## Abstract

**Introduction:**

In pulmonary arterial hypertension (PAH), it remains unclear whether absolute haemodynamic values or treatment-induced changes are more relevant to subsequent changes in clinical status. As risk classifications are increasingly used to guide clinical management, we evaluated whether pulmonary vascular resistance (PVR) at baseline, after 4 months of therapy, or treatment-induced change better explains risk change.

**Methods:**

Among 104 screened PAH patients, 69 were included retrospectively. The primary outcome was change in clinical status, defined as a shift in ESC/ERS 4-strata risk classification from baseline to 15 months. Haemodynamics were evaluated via right heart catheterization before and after 4 months of pharmacotherapy. Linear regression examined associations of baseline PVR, 4-month PVR, and ΔPVR with risk change. ROC analyses explored the ability of PVR (baseline, 4-month, and Δ), ΔRVEDP, and ΔCI to discriminate between risk stabilization and worsening.

**Results:**

Risk classification and PVR decreased significantly following treatment (*p*<0.001). ΔPVR had the highest partial explained variance for risk change (R^2^=0.118, *p*=0.003), followed by baseline PVR (R^2^=0.098, *p*=0.013). Four-month PVR did not significantly predict risk classification change. In ROC analyses, only ΔPVR significantly discriminated risk stabilization (AUC=0.75, *p*<0.001). Bootstrapping and permutation confirmed the robustness of these findings.

**Conclusions:**

The change in PVR after 4 months of treatment outperformed baseline and 4-month PVR, but also other dynamic measures (ΔRVEDP and ΔCI), in explaining changes in 4-strata risk classification and identifying risk stabilization. These findings suggest that ΔPVR provides complementary insight when interpreting serial haemodynamic evaluations in the context of early treatment response in PAH.

Despite therapeutic advances, pulmonary arterial hypertension (PAH) remains life-threatening, with a 3-year mortality rate of 21%.^1^, ^2^ PAH involves pulmonary arterial remodeling, leading to increased pulmonary vascular resistance (PVR) and ultimately right ventricular (RV) failure—the principal determinant of prognosis.^3^, ^4^ PAH-specific pharmacotherapy aims to lower PVR and stabilize RV function. These treatment-induced reductions in PVR are assumed to play a key role in improving clinical outcomes, including functional status and survival.^3^, ^4^, ^5^, ^6^, ^7^, ^8^, ^9^, ^10^ However, the precise relationship between haemodynamics and clinical improvement remains debated.^11^

Previous studies examining the prognostic value of absolute haemodynamic values at baseline or follow-up in PAH have yielded conflicting findings. Some studies link baseline or follow-up haemodynamics to clinical outcomes, while others stress the role of RV functional adaptation and treatment-induced changes in afterload.^12^, ^13^, ^14^, ^15^, ^16^ In line with this, a recent International Society for Heart and Lung Transplantation consensus statement on risk stratification in PAH emphasizes the prognostic value of haemodynamic indices at baseline and follow-up, while acknowledging uncertainty regarding the optimal target for longitudinal interpretation of treatment response.^17^ This highlights the need to study absolute PVR before and after pharmacotherapy and treatment-induced change in PVR as markers of disease trajectory and treatment response. However, varying outcome measures contribute to inconsistency.^11^, ^16^, ^18^, ^19^ For example, most studies relate PVR to a single marker of exercise tolerance (e.g., 6-minute walking distance), rather than current risk-based treatment targets (e.g., the 4-strata model of the European Society of Cardiology (ESC)/European Respiratory Society (ERS)).^3^, ^11^

This retrospective study investigates the relationship between PVR at baseline, after 4 months of PAH-specific pharmacotherapy, and the corresponding change (i.e., ΔPVR) in relation to clinical risk change according to the ESC/ERS 4-strata model at 15-month follow-up in patients with PAH. We hypothesize that early pharmacotherapy-driven change in PVR outperforms absolute PVR levels at baseline or following 4 months of treatment in explaining 15-month clinical risk change, given that changes in afterload promote RV remodeling and functional improvement. Understanding how haemodynamic assessments should be interpreted in the context of change in risk following treatment may inform the interpretation of serial right heart catheterization (RHC) in PAH. As a secondary objective, we explore whether changes in cardiac index and RV end-diastolic pressure, both markers of RV function, are associated with stabilization of clinical risk. Alongside PVR, studying changes in these markers may help understand how haemodynamics relate to treatment-associated risk change in PAH.

## Methods

### Population and study design

This retrospective, single-center observational cohort study included 69 adult patients identified through medical record screening at Royal United Hospitals Bath (Combe Park, Bath, United Kingdom) between November 2008 and July 2022. Inclusion criteria were (1) aged ≥ 18 years, (2) confirmed pulmonary hypertension World Health Organization (WHO) Group 1 (i.e., PAH, all etiologies) through RHC according to contemporary guidelines, (3) received PAH-specific pharmacotherapy, and (4) clinical follow-up data available in year 2 after treatment initiation. Exclusion criteria were (1) clinical follow-up of <1 year, (2) vasoreactive PAH, (3) lung transplant or mortality if precluding clinical follow-up in year 2, and (4) no follow-up RHC within 11 months after treatment initiation. Ethics approval and informed consent were waived by the local Medical Ethics Review Committee (record #2023–16669).

### Measurements

Data were extracted from electronic medical records of the Pulmonary Hypertension Service at Royal United Hospitals Bath. Baseline characteristics (age, sex, height, weight, smoking status, medical history, PAH etiology, baseline transthoracic echocardiographic (TTE) recordings, and PAH-specific medication initiated after the first RHC and at least 1 week before the second RHC) were collected and body mass index (BMI) and body surface area (BSA) using the Du Bois formula were calculated to characterize the patient population. To provide additional clinical characterization of the cohort, a limited set of key TTE parameters was assessed, including peak tricuspid regurgitation velocity (TRV), tricuspid annular plane systolic excursion (TAPSE), and the TAPSE/systolic pulmonary arterial pressure (sPAP) ratio. Estimated sPAP was derived using the Bernoulli equation. These parameters are presented for descriptive purposes only and were not included in the primary analyses. TTE images were analyzed by a single UK-registered sonographer (CW) following societal guidelines.^3^, ^20^, ^21^

The primary outcome was the change in ESC/ERS 4-strata risk score (i.e., Δrisk) between baseline and after a median follow-up of 15 months. A change-based risk endpoint was selected as a proxy of long-term treatment response, as absolute risk categories do not account for baseline disease severity and therefore may be less suited to quantify treatment-associated effects. The assigned risk score depended on the average score based on assigned points (1-4), for 6-minute walking distance (6MWD), WHO functional class (WHO-FC), and N-terminal pro-B-type natriuretic peptide (NT-proBNP) levels. A missing value for 1 parameter was allowed.^3^ Risk scores were not rounded. Changes were calculated between baseline and 15-month follow-up. Given the discrete but approximately continuous distribution of Δrisk (19 possible values), Δrisk was analyzed as a continuous outcome using linear regression.

Secondary outcomes included cardiopulmonary haemodynamics measured through RHC at baseline and after a median of 4 months of treatment (e.g., 4-month PVR), reflecting early reassessment after treatment initiation. RHC was performed in accordance with European guidelines,^3^ using Swan-Ganz catheters calibrated in line with standard clinical practice, with the patient in a supine position. Baseline RHCs confirmed the diagnosis of PAH, while follow-up RHCs were conducted to assess treatment response. Cardiac output (CO) was measured using the thermodilution technique, and cardiac index (CI) was calculated as CO divided by BSA. For cases where BSA data were unavailable, CI values were taken from clinical RHC reports. Invasive cardiopulmonary pressures, including mean pulmonary arterial pressure (mPAP), pulmonary arterial wedge pressure (PAWP), and RV end-diastolic pressure (RVEDP), were recorded. PVR was calculated as (mPAP - PAWP)/CO. Haemodynamic changes were calculated between baseline and 4 months of treatment and are expressed as absolute changes.

### Statistics

Baseline characteristics were reported as mean (M) ± standard deviation (SD), while continuous haemodynamic and risk model parameters were summarized as median ± interquartile range (IQR). Categorical variables were presented as frequencies (n) and percentages (%). Normality of continuous outcome variables was assessed using Shapiro-Wilk tests. Within-group differences between baseline and 4 months of treatment (haemodynamic parameters) or 15-month follow-up (risk model parameters) were evaluated using Wilcoxon signed-rank tests, given that several key outcome variables were not normally distributed. The Stuart Maxwell test was used to compare the distribution of risk class and WHO-FC at baseline and follow-up.

Our primary objective was to test the hypothesis that ΔPVR is superior in predicting changes in clinical risk score at follow-up (median 15 months), compared to PVR at baseline or after 4 months of pharmacotherapy. Therefore, we performed multiple linear regression with Δrisk as the dependent variable, comparing 3 key PVR-variables (baseline PVR, 4-month PVR, ΔPVR) while adjusting for age and sex. For each PVR-based predictor, we evaluated a model including only main effect, but also a model including an interaction term between the PVR-variable and age given the known age-related variation in treatment response in PAH.^22^ To assess potential interdependence between predictors, Spearman’s rank correlations were calculated between absolute PVR values and ΔPVR alongside regression modeling. Model assumptions were checked and confirmed using residual plots, histograms, Q-Q plots, VIF (generalized for interaction models) and Breusch-Pagan tests.

To assess overall model performance, we reported adjusted R^2^ values of the full models, reflecting the proportion of explained variance, corrected for the number of predictors.

Finally, to quantify the predictive strength of individual predictors, we calculated the partial explained variance (partial R^2^), defined as the proportion of variance uniquely explained by the predictor, or equivalently, the decrease in model performance when the predictor is omitted. To improve robustness against outliers and to assess model stability, partial R^2^ values were derived through bootstrapping (10,000 resamples) and were reported as median with IQR.

As a secondary, exploratory objective, we assessed the ability of haemodynamic parameters to discriminate clinical risk stabilization at follow-up (i.e., Δrisk≤0). Receiver operating characteristic (ROC) curves were generated for baseline PVR, 4-month PVR, ΔPVR, ΔRVEDP, and ΔCI to assess and compare their discriminative performance. RVEDP was included as a marker of end-diastolic intraventricular pressure to represent RV preload and compliance in the context of early treatment-related haemodynamic changes. Area under the curve (AUC) was calculated for each predictor, and DeLong tests were performed to assess whether AUCs deviated from 0.5, indicating discriminative ability.

Significance was determined at α<0.05. Data processing and descriptives were performed using SPSS Statistics 29 (IBM Corp, Armonk, NY, USA), while visualizations and analyses were conducted using RStudio 2024.04 (PBC, Boston, MA, USA) with the *car*,^23^ *lmtest*,^24^ *pROC*,^25^ and *rsq*^26^ packages.

## Results

### Baseline characteristics

**Figure 1:**
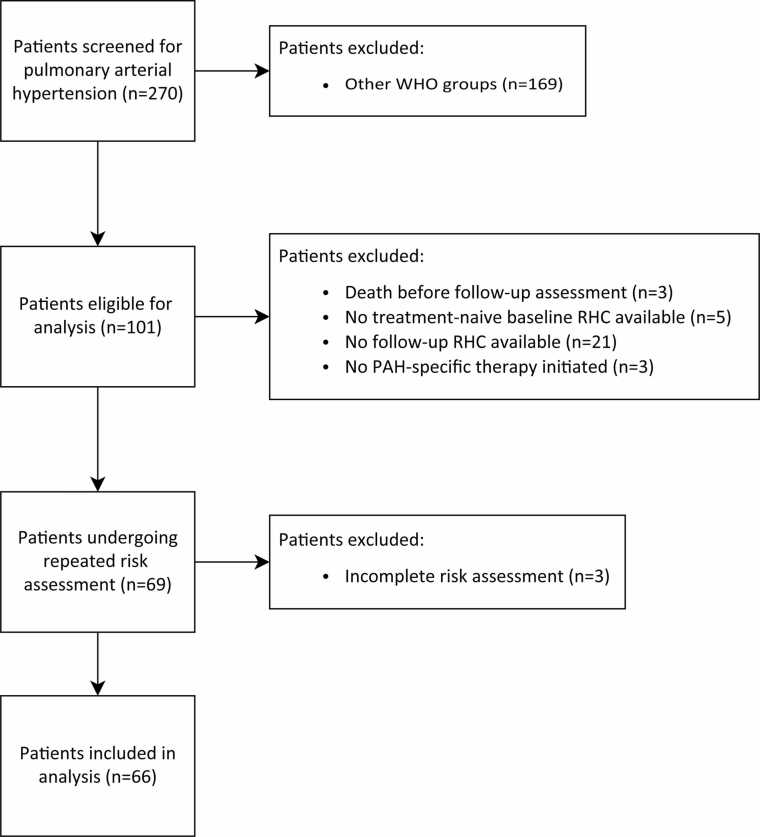
Patient selection and inclusion at Royal United Hospitals Bath (Bath, United Kingdom). Patients were screened for pulmonary arterial hypertension and included based on predefined criteria. Individuals with available baseline and follow-up right heart catheterization and risk assessment were included in the final analysis. PAH, pulmonary arterial hypertension; RHC, right heart catheterization; WHO, World Health Organisation

Of the 104 screened patients with PAH, 69 met the eligibility criteria (Figure 1). After excluding those with incomplete risk scores, the final cohort comprised 66 patients, including 51 females (77%) and 15 males (23%) aged 62±15 years. BMI at diagnosis was 27.2±5.3 kg/m^2^. Half of the patients had never smoked. Medical history included hypertension (32%), coronary artery disease (14%), diabetes mellitus (15%), chronic kidney disease (15%), COPD (6%), and prior pulmonary embolism (9%). The majority of patients were diagnosed with connective tissue disease-associated PAH (CTD-PAH) (70%) or idiopathic PAH (26%). Mean baseline values of TRV, TAPSE, and TAPSE/sPAP ratio were consistent with echocardiographic signs of pulmonary hypertension according to ESC/ERS guidelines.^3^ Most patients received initial combination therapy with an endothelin receptor antagonist (ERA) and a phosphodiesterase-5 inhibitor (PDE5i) (65%), while 29% received PDE5i monotherapy, 5% received ERA monotherapy, and one patient received a PDE5i-prostacyclin analog combination. Two patients were switched between different ERAs and PDE5is due to side effects. The median time from baseline RHC to repeat RHC was 4 (3-6) months. Population characteristics are presented in Table 1.

**Table 1:** Baseline Characteristics

**Variable**	**Value (n = 66)**	**Missing (n (%))**
*Demographics*		
Age (y)	62 (15)	-
Female sex (n (%))	51 (77)	-
*Anthropometrics*		
Height (m)	1.65 (0.09)	12 (18)
Weight (kg)	74 (16)	12 (18)
BMI (kg/m^2^)	27.2 (5.3)	12 (18)
BSA (m^2^)	1.8 (0.2)	12 (18)
*Smoking history (n (%))*		
Current tobacco use	5 (8)	6 (9)
Former tobacco use	25 (42)	6 (9)
*Medical history (n (%))*		
Hypertension	21 (32)	-
Coronary artery disease	9 (14)	-
Cardiomyopathy	1 (2)	-
Diabetes mellitus	10 (15)	-
Chronic kidney disease	10 (15)	-
COPD	4 (6)	-
Pulmonary embolism	6 (9)	-
*PAH etiology (n (%))*		
Connective tissue disease	46 (70)	-
Idiopathic	17 (26)	-
Congenital heart disease	1 (2)	-
Connective tissue disease + congenital heart disease	1 (2)	-
Connective tissue disease + drug/toxin induced	1 (2)	-
*Echocardiography*		
TRV (m/s)	3.8 (0.8)	12 (18)
TAPSE (mm)	17 (4)	10 (15)
TAPSE/sPAP ratio (mm/mmHg)	0.29 (0.17)	21 (32)
*Invasive haemodynamics (RHC)*		
RAP (mmHg)	8 (6-12)	-
RVEDP (mmHg)	12 (9-16)	2 (3)
sPAP (mmHg)	74 (58-82)	3 (5)
dPAP (mmHg)	28 (23-34)	3 (5)
mPAP (mmHg)	45 (36-53)	-
PAWP (mmHg)	10 (9-13)	-
CI (L/min/m^2^)	2.3 (2.0-2.6)	1 (2)
PVR (WU)	8.3 (6.2-11.2)	-
*PAH Medication (n (%))*		
ERA	3 (5)	-
PDE5i	19 (29)	-
ERA + PDE5i	43 (65)	-
PDE5i + PCA	1 (2)	-

Baseline characteristics and medication use of the study population. Variables are presented as mean (SD), unless indicated otherwise. Medication use reflects prescriptions ≥ 1 week prior to the second right heart catheterization. BMI, body mass index; BSA, body surface area; CI, cardiac index; dPAP, diastolic pulmonary arterial pressure; ERA, endothelin receptor antagonist; mPAP, mean pulmonary arterial pressure; PAH, pulmonary arterial hypertension; PAWP, pulmonary arterial wedge pressure; PCA, prostacyclin analog; PDE5i, phosphodiesterase-5 inhibitor; PVR, pulmonary vascular resistance; RAP, right atrial pressure; RHC, right heart catheterization; RVEDP, right ventricular end-diastolic pressure; sPAP, systolic pulmonary arterial pressure; TAPSE, tricuspid annular plane systolic excursion; TRV, peak tricuspid regurgitation velocity

### Haemodynamics and risk score at baseline and after treatment

**Figure 2:**
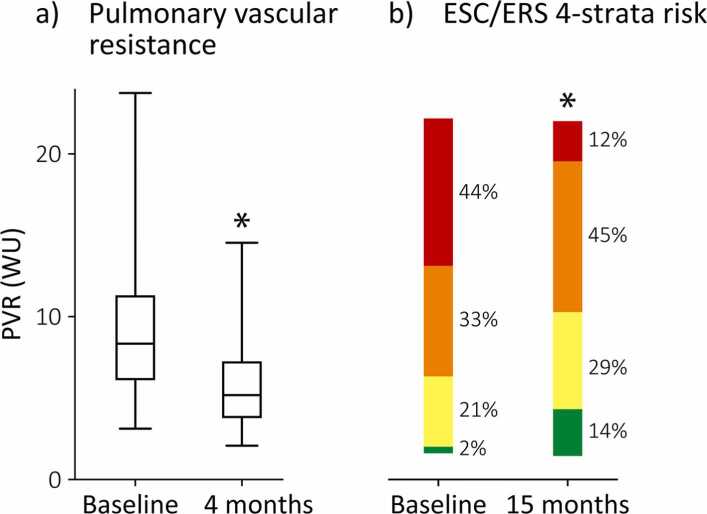
Median ± interquartile range of pulmonary vascular resistance (WU) and the distribution of ESC/ERS 4-strata risk classification (low (green), intermediate-low (yellow), intermediate-high (orange), and high (red) risk) at baseline and at a median follow-up of 4 and 15 months, respectively (n = 66). Boxes represent interquartile range and whiskers represent 2.5th and 97.5th percentiles. ESC/ERS, European Society of Cardiology/European Respiratory Society; PVR, pulmonary vascular resistance; WU, Wood units. * *p*<.001 for comparisons between baseline and 4-month or 15-month values

Following 4 months of treatment, we found significant changes in all haemodynamic parameters except for RVEDP and PAWP. We observed a decline in PVR from 8.3 Wood units (WU) at baseline to 5.2 WU after 4 months of treatment (*p*<0.001, Figure 2). Invasive mPAP declined from 45 mmHg to 37 mmHg (*p*<0.001), and CI increased from 2.3 to 2.9 L/min/m^2^ (*p*<0.001). RVEDP was 12 mmHg at baseline and 11 mmHg at 4 months (*p*>0.05, Table 2).

**Table 2:** Effect of Treatment on Haemodynamics and ESC/ERS 4-strata Risk

**Invasive haemodynamics**			**ESC/ERS 4-strata risk parameters**					
**Outcome**	**Value (n = 66)**	**Missing (n (%))**	**Outcome**	**Value (n = 66)**				**Missing (n (%))**
RAP (mmHg)			ESC/ERS 4-strata risk					
Baseline	8 (6-12)	-	Baseline	3.0 (2.3-3.7)				-
4-month*	7 (5-8)	-	4-month**	2.4 (2.0-3.0)				16 (24)
Δ	−1 (−4 to 1)	-	15-month**	2.3 (1.7-2.7)				-
RVEDP (mmHg)			Δ	−0.5 (−1.0 to 0.0)				-
Baseline	12 (9-16)	2 (3)	WHO-FC (frequencies)	**I**	**II**	**III**	**IV**	
4-month	11 (9-13)	-	Baseline (n)	-	2	60	3	1 (2)
Δ	−1 (−4 to 2)	2 (3)	4-month		7	54		5 (8)
sPAP (mmHg)			15-month (n)*	1	12	52	1	-
Baseline	74 (58-82)	3 (5)	6MWD (m)					
4-month**	63 (50-76)	1 (2)	Baseline	282 (128-392)				5 (8)
Δ	−9 (−17 to 1)	3 (5)	4-month	360 (240-427)				52 (26)
dPAP (mmHg)			15-month**	347 (226-438)				2 (3)
Baseline	28 (23-34)	3 (5)	Δ	40 (−6 to 125)				7 (11)
4-month**	22 (18-28)	1 (2)	NT-proBNP (pg/mL)					
Δ	−6 (−9 to −1)	3 (5)	Baseline	961 (236-3027)				1 (2)
mPAP (mmHg)			4-month**	390 (170-753)				16 (24)
Baseline	45 (36-53)	-	15-month**	305 (107-793)				3 (5)
4-month**	37 (31-46)	-	Δ	−364 (−1927 to −8)				4 (6)
Δ	−7 (−12 to 0)	-						
PAWP (mmHg)								
Baseline	10 (9-13)	-						
4-month	11 (9-13)	-						
Δ	0 (−2 to 2)	-						
CI (L/min/m^2^)								
Baseline	2.3 (2.0-2.6)	1 (2)						
4-month**	2.9 (2.5-3.2)	1 (2)						
Δ	0.6 (0.2-1.0)	2 (3)						
PVR (WU)								
Baseline	8.3 (6.2-11.2)	-						
4-month**	5.2 (3.8-7.2)	-						
Δ	−3.1 (−4.9 to −1.2)	-						

Haemodynamic parameters at baseline and 4 months (left), and risk score parameters at baseline, 4-months and 15-month follow-up (right). Risk parameters at 4 months were based on measurements obtained within 1 month of the 4-month RHC. For risk parameters, changes (Δ) are calculated between baseline and 15-month values. Outcomes are presented as median (interquartile range), unless indicated otherwise. Asterisks indicate statistically significant differences compared with baseline values. 6MWD, six-minute walking distance; CI, cardiac index; dPAP, diastolic pulmonary arterial pressure; ESC/ERS, European Society of Cardiology/European Respiratory Society; mPAP, mean pulmonary arterial pressure; NT-proBNP, N-terminal pro B-type natriuretic peptide; PAWP, pulmonary arterial wedge pressure; PVR, pulmonary vascular resistance; RAP, right atrial pressure; RHC, right heart catheterization; RVEDP, right ventricular end-diastolic pressure; sPAP, systolic pulmonary arterial pressure; WHO-FC, World Health Organization functional class; WU, wood units

* *p*<.05;

** *p*<.001.

Regarding the ESC/ERS 4-strata risk model at baseline, 2% of patients were classified as low risk, 21% as intermediate-low, 33% as intermediate-high and 44% as high risk. At 15-month follow-up, a significant shift towards lower risk strata was observed (*p*<0.001, Figure 2). The median 4-strata risk score decreased from 3.0 to 2.3 at follow-up (*p*<0.001, Table 2). Among the individual risk parameters, the distribution of WHO-FC showed a significant shift towards lower classes at follow-up (*p*<0.01); 6MWD increased from 282 m to 347 m (*p*<0.001); and NT-proBNP levels decreased from 961 pg/mL to 305 pg/mL (*p*<0.001).

### PVR in relation to clinical risk reduction

**Figure 3:**
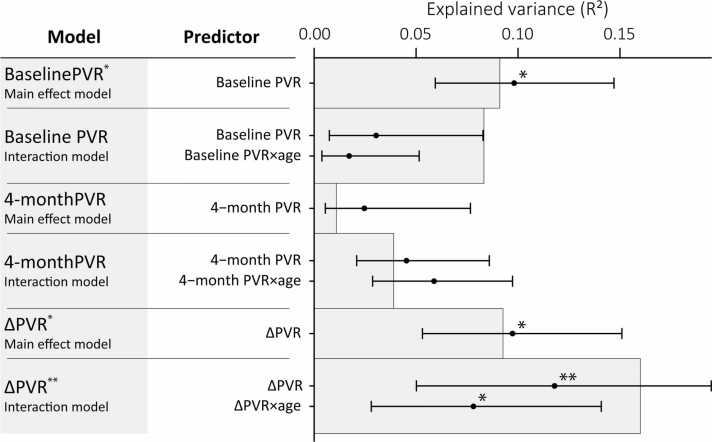
Predictor and model-level explained variance in predicting 15-month change in ESC/ERS 4-strata risk score (n = 66). Predictors and models are grouped on the Y-axis. The X-axis shows the explained variance (R^2^). Dots represent median bootstrapped partial R^2^ for individual predictors, with error bars marking the interquartile range. The gray-colored bands indicate the adjusted R^2^ of the full models. ESC/ERS, European Society of Cardiology/European Respiratory Society; PVR, pulmonary vascular resistance. * *p*<.05; ^**^p<.01

In predicting the 15-month change in risk score, the baseline PVR main effect model showed significant explanatory power (adjusted R^2^=0.091, *p*=0.030), with baseline PVR significantly contributing to the model (median partial R^2^=0.098, *p*=0.013; Figure 3, Supplementary Tables A1–A12). Specifically, a higher baseline PVR corresponded with a greater risk decline. For 4-month PVR data, neither the main effect nor the interaction model showed a significant relation with the change in clinical risk score.

Both ΔPVR models (main and interaction) demonstrated significant explanatory power (adjusted R^2^=0.093 and 0.160; *p*=0.029 and 0.005, respectively). ΔPVR significantly contributed to both the main effect model and the interaction model (median partial R^2^=0.097 and 0.118, *p*=0.012 and 0.003, respectively; Fig. 3, Supplementary Tables S1–S12), with a larger decrease in PVR associated with larger risk-decline. The ΔPVR×age interaction was significant (median partial R^2^=0.078, *p*=0.017). A Spearman’s rank correlation between baseline PVR and ΔPVR was significant (r=-0.729, *p*<0.001), whereas 4-month PVR and ΔPVR were not significantly associated (r=-0.147, *p*=0.120).

A post hoc permutation test was used to assess whether the ΔPVR model significantly outperformed other models, and this was confirmed (Supplementary Methods, Supplementary Figure S1).

### ROC-curves: haemodynamics and risk stabilization

**Figure 4:**
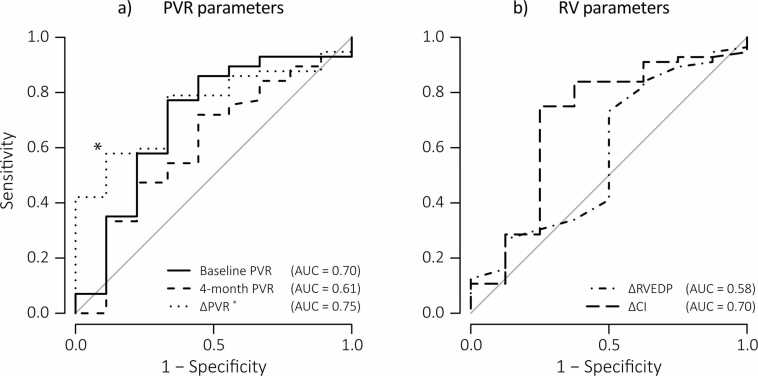
ROC-curves indicating discriminative performance of baseline PVR, 4-month PVR, and ΔPVR (panel a) and ΔRVEDP and ΔCI (panel b) for ESC/ERS 4-strata risk stabilization at 15-month follow-up (Δrisk≤0; n = 66). AUC, area under the curve; CI, cardiac index; ESC/ERS, European Society of Cardiology/European Respiratory Society; PVR, pulmonary vascular resistance; RV, right ventricle; RVEDP, right ventricular end-diastolic pressure. * *p*<.001

ΔPVR significantly discriminated risk stabilization at 15-month follow-up (AUC=0.75, *p*<0.001) whereas baseline and 4-month PVR did not (AUC=0.70 and 0.61; *p*=0.062 and 0.306, respectively; Figure 4). Additional RV parameters were not discriminative: ΔCI yielded an AUC of 0.70 (*p*=0.091), and ΔRVEDP an AUC of 0.58 (*p*=0.520). Given an imbalance in outcome distribution (n = 57 risk stabilization, n = 9 worsening), bootstrapping was performed using 10,000 resamples, suggesting that the AUC estimates were relatively stable (results not shown).

## Discussion

The purpose of our study was to examine the relationship between baseline PVR, 4-month PVR, or the change in PVR between these time points and the change in ESC/ERS 4-strata risk at 15 months after treatment initiation in patients with PAH. Our analysis revealed the following key findings. First, both a higher baseline PVR and a larger decrease in PVR, but not PVR at 4 months, were associated with larger improvements in risk stratification at 15-month follow-up. Second, we found that the change in PVR consistently outperforms baseline PVR in explaining variation in risk reduction in patients with PAH. These observations were independent of age and sex. Third, ROC curve analysis demonstrated that the change in PVR, but not absolute PVR at baseline or 4 months, significantly predicts stabilization of clinical risk. Finally, 4-month changes in additional haemodynamic measures (i.e., ΔCI and ΔRVEDP) had no significant discriminative value for risk stabilization. Taken together, these findings suggest that changes in PVR, rather than PVR at single time points (baseline or 4 months), are informative for interpreting early treatment response and are associated with subsequent changes in ESC/ERS 4-strata risk classification following pharmacotherapy.

We found that ΔPVR emerged as the strongest invasive haemodynamic correlate of clinical risk change at 15-month follow-up, supporting our hypothesis that PVR reduction following therapy outperforms both baseline and 4-month PVR. Importantly, ΔPVR remained significant after accounting for a potential ΔPVR-age interaction and retained superior discriminative power in ROC curve analysis. Physiologically, this suggests that early ΔPVR effectively captures dynamic, treatment-induced pulmonary vascular adaptation. While prior studies have focused on the prognostic value of haemodynamic parameters at single time points and, to a lesser extent, changes in PVR, none have directly compared baseline, follow-up and ΔPVR in relation to longitudinal changes in clinical risk.^11^ Although these studies suggest prognostic value for absolute PVR measurements at follow-up, our findings provide novel insight by identifying ΔPVR—rather than baseline or follow-up values—as the only parameter that is consistently associated with ESC/ERS 4-strata risk classification improvement.

Beyond the magnitude of risk change, however, the primary therapeutic goal in PAH is to achieve and maintain a low-risk status. This raises the question of whether absolute haemodynamic targets, while not reflecting the magnitude of risk modification, may still be relevant for predicting attainment of a low-risk status. To address this, we conducted an exploratory post hoc analysis assessing the association between achieving a 4-month PVR threshold of <6 WU and attaining a low-risk status at 15 months. No such association was observed in our cohort, albeit limited by the small number of patients reaching low-risk status. Previous work by Dardi et al. provides important context by reporting that haemodynamic cut-offs at follow-up are associated with mortality and clinical worsening, but that these cut-offs do not improve mortality prediction compared with current risk stratification tools.^27^ Together, these findings suggest that absolute PVR at follow-up and risk scores may reflect partly overlapping information, which could limit the ability of early PVR thresholds to discriminate later risk status. Instead, absolute PVR thresholds may primarily reflect disease severity and prognosis, whereas changes in PVR were associated with changes in risk over time, supporting the potential relevance of ΔPVR as a marker of the clinical trajectory under treatment.

Interestingly, we identified a significant interaction between ΔPVR and age, indicating that the link between ΔPVR and risk change diminishes with older age. This observation suggests that changes in pulmonary haemodynamics show a weaker association with subsequent risk change in older patients compared to younger individuals. One potential explanation for this observation is that older age is associated with an attenuated potential for reverse remodeling of the RV and pulmonary arterial changes following treatment, which may in part reflect age-related myocardial fibrosis and reduced ventricular compliance. In addition, recent work has demonstrated that age-related comorbidities may affect components of non-invasive risk scores (e.g., functional capacity and biomarkers), resulting in higher risk classification independent of haemodynamic severity.^28^ This aligns with previous reports highlighting that older age is associated with attenuated treatment response and less favorable clinical outcomes in idiopathic PAH.^22^ Clinically, early changes in PVR should be interpreted with caution in older patients, as early reductions in afterload may less accurately reflect the actual potential for clinical risk reduction.

We observed a positive association between baseline PVR and risk reduction, which means that patients with higher baseline PVR experienced greater risk reduction. This may seem counterintuitive as high baseline PVR typically signals more advanced disease. One potential explanation for our observation is that a higher initial afterload mathematically allows for larger treatment-induced reductions. This may primarily reflect that patients with higher baseline pulmonary vascular resistance experience greater reductions in right ventricular afterload during treatment, leading to more pronounced improvements in cardiac output and downstream components of the risk score, including functional capacity and biomarkers. Prior reports have indeed suggested that the clinical benefit of afterload reduction depends on RV contractile reserve and preserved ventriculoarterial coupling rather than baseline PVR.^15^ To support this, the change in PVR outperformed the absolute baseline PVR value to predict risk progression. A statistical consideration in this context is regression-to-the-mean, which may have contributed to variability in repeated measurements, particularly in patients with more extreme baseline values. However, this statistical phenomenon primarily affects within-parameter variability over repeated assessments and may therefore have a smaller potential impact on the observed association between baseline PVR and subsequent changes in clinical risk. Our findings may help reconcile previous conflicting observations regarding haemodynamic responses in patients with higher baseline afterload.^12^, ^13^, ^14^ In contrast to baseline PVR, we found that 4-month PVR did not predict risk improvement in our cohort. While PVR at follow-up reflects residual afterload, it may not capture the extent of RV unloading or recovery potential. Taken together, our findings suggest that absolute measures of afterload, whether at baseline or follow-up, have limited relevance for assessing risk change. In contrast, ΔPVR appears to capture a specific component of treatment-related haemodynamic improvement, although clinical risk improvement is likely influenced by multiple additional haemodynamic and non-haemodynamic factors.

We explored the ability of haemodynamic parameters to discriminate clinical risk stabilization. Among the variables tested (i.e., baseline PVR, 4-month PVR, ΔPVR, ΔCI, and ΔRVEDP), only ΔPVR demonstrated discriminative value in ROC curve analyses. This may indicate that changes in RV functional parameters reflect downstream adaptation rather than serving as early markers of treatment-associated risk change. It is important to note that CI and RVEDP may not fully capture RV performance, and other parameters of RV function (e.g., stroke volume index and RV end-systolic elastance) may improve early assessment of risk change alongside ∆PVR. Taken together, these findings suggest that not all haemodynamic changes are equally informative for identifying patients who achieve subsequent stabilization of clinical risk.

### Limitations

Strengths of our study include the use of repeated invasive haemodynamic evaluations, the direct comparison of absolute and dynamic PVR indices for assessing subsequent changes in ESC/ERS 4-strata risk classification, and the real-world applicability of our findings. Some limitations must be acknowledged. First, the retrospective single-center design lacks external validation, and the limited sample size may affect generalizability. However, the internal validity and procedural consistency of this setup strengthen clinical interpretability. Second, the use of ESC/ERS 4-strata risk classification may introduce limited granularity and a ceiling effect and, consequently, a skewed data distribution. The latter may challenge the robustness of linear models. Therefore, we repeated our analysis using non-linear approaches, which confirmed our main results. Third, although changes in risk scores have been proposed for evaluating treatment response, they should be interpreted with caution when used to infer treatment response, as these scores were developed for prognostic stratification and are not validated surrogate endpoints. Fourth, our cohort included a substantial proportion of CTD-PAH, which presents with distinct clinical and haemodynamic characteristics. In particular, patients with CTD-PAH may exhibit a less pronounced improvement in clinical risk despite haemodynamic response, potentially attenuating the observed associations. This cohort composition reflects the referral pattern to a tertiary center and may limit generalizability to other PAH subgroups. The exclusion of patients who died or underwent lung transplantation prior to follow-up may have introduced a degree of survivor bias, although the number of such cases was limited (n=3). In addition, the requirement for repeat right heart catheterization may have contributed to the exclusion of patients with a more favorable clinical profile. Adjustment for comorbidities and comparison with other risk models (e.g., REVEAL, COMPERA) were precluded by the study design. Though age and sex were adjusted for, residual confounding cannot be excluded. Finally, due to our retrospective design, we were unable to control for clinical events (e.g., hospitalization) and pharmacotherapeutic adjustments following the second RHC. Pharmacotherapeutic adjustments likely occurred in patients with limited clinical improvement and/or haemodynamic response. As such factors may have affected clinical status independent of early haemodynamic change, this actually attenuated the observed relation.

In conclusion, the main finding from our retrospective cohort study is that the change in pulmonary vascular resistance (ΔPVR) during the first 4 months of pharmacotherapy was the strongest and most consistent invasive haemodynamic marker of changes in ESC/ERS 4-strata risk stratification at 15-month follow-up in patients with PAH. Notably, PVR after 4 months of pharmacotherapy was not associated with risk change. These findings suggest that dynamic changes in PVR offer greater support for interpreting serial haemodynamic evaluations in relation to risk change. From a clinical perspective, treatment-associated shifts in afterload may be more closely aligned with underlying disease adaptation, supporting the notion that early RV unloading is pivotal in achieving lower risk status in PAH.

## Financial support

The position of SD was funded through an institutional grant from J&J Innovative Medicine to Radboudumc (grant number 67896049PUH4007). The funding source had no role in the design, interpretation, or reporting of the work.

## Declaration of Competing Interest

The authors declare the following financial interests/personal relationships which may be considered as potential competing interests: Jay Suntharalingam reports a relationship with MSD that includes consulting or advisory. Jay Suntharalingam reports a relationship with Ferrer that includes consulting or advisory. Jay Suntharalingam reports a relationship with Janssen Pharmaceuticals Inc that includes consulting or advisory. Jay Suntharalingam reports a relationship with Janssen Pharmaceuticals Inc that includes speaking and lecture fees. Jay Suntharalingam reports a relationship with Ferrer that includes travel reimbursement. Robert V. MacKenzie Ross reports a relationship with Janssen Pharmaceuticals Inc that includes travel reimbursement. Arie. P.J. van Dijk reports a relationship with Janssen Pharmaceuticals Inc that includes consulting or advisory and speaking and lecture fees. David Oxborough reports a relationship with Pfizer that includes speaking and lecture fees. David Oxborough serves as Chair of Education Committee and as a Trustee and Council member of the British Society of Echocardiography (BSE). The other authors, declare that they have no known competing financial interests or personal relationships that could have appeared to influence the work reported in this paper.

## Footnotes

Appendix A Supplementary data associated with this article can be found in the online version at doi:10.1016/j.jhlto.2026.100599.
