# Supplementary material for: Absolute versus change in pulmonary vascular resistance in relation to European Society of Cardiology/European Respiratory Society risk change in pulmonary arterial hypertension
Source: JHLT Open. 2026 May 20;13:100599. doi: 10.1016/j.jhlto.2026.100599 (PMC13262166; doi:10.1016/j.jhlto.2026.100599)
Supplement: Supplementary file 1 — Supplementary material [file mmc1.docx]

**Supplementary material**

**Absolute versus change in pulmonary vascular resistance in relation to European Society of Cardiology/European Respiratory Society risk change in pulmonary arterial hypertension**

Stijn C.M. Donker, MD, Christopher J.B. Wild, Daniel X. Augustine, MD, Jay Suntharalingam, MD, Robert V. MacKenzie Ross, MD, Arie P.J. van Dijk, MD PhD, Joseph D. Maxwell, PhD, David Oxborough, PhD, Dick H.J. Thijssen, PhD

**Supplementary Methods. Comparison of two best performing models through permutation test**

**Supplementary Figure S1. Model performance comparison of the two best models**

**Supplementary Tables S1-S12. Overview of model performance and predictor contributions**

Supplementary Methods. Comparison of two best performing models through permutation test

*Statistics*

To determine whether the observed difference in explained variance (i.e., adjusted R^2^) between models was statistically significant, we employed permutation testing. This method involves randomly reassigning the outcome variable (i.e., Δrisk) across participants, while keeping the predictor variables fixed. This procedure disrupts any true association between predictors and outcome, simulating a scenario where model performance arises purely by chance. For each permuted dataset, adjusted R^2^ was calculated for the two best-performing models. Repeating this procedure 10,000 times yielded a null distribution of adjusted R^2^ differences between these models, under the assumption that neither model has real predictive value.

We then compared the observed adjusted R^2^ difference between the two originally selected models – each representing a different PVR category (i.e., baseline, 4-months or Δ) – to the null distribution obtained through permutation testing. Model selection was performed as follows: first, the best-performing overall model was identified based on largest adjusted R^2^ and the presence of at least one significant PVR-term. Second, the best-performing model from one of the remaining PVR categories (i.e., not the category of the top model) was selected using the same criteria (i.e., highest adjusted R^2^ and at least one significant PVR-term). If the observed difference between these models’ adjusted R^2^ exceeded the 95^th^ percentile of the null distribution, it was considered statistically significant (*p*<0.05).

If initial testing using 1,000 permutations suggested significance (*p*<0.05) , a full test with 10,000 permutations was performed to confirm robustness. Multiple independent permutation sequences were run, yielding consistent results.

*Results*

In addition to the best-performing ΔPVR interaction model (adjusted R^2^=0.160, *p=0.005*), the baseline PVR main effect model was selected as the second-best-performing model from the other PVR categories (adjusted R^2^=0.091, *p*=0.030). Model comparison based on AIC and BIC values resulted in identical model ranking *(Supplementary Tables S1-S12*). The observed adjusted R^2^ difference (ΔR^2^=0.069) exceeded the 95% range of the null distribution (ΔR^2^=0.000–0.058), confirming statistical significance of the observed difference (*p*=0.033). The results of the permutation test are illustrated in *Supplementary Figure S1.*

**Supplementary Figure S1. Model performance comparison of the two best models**

**
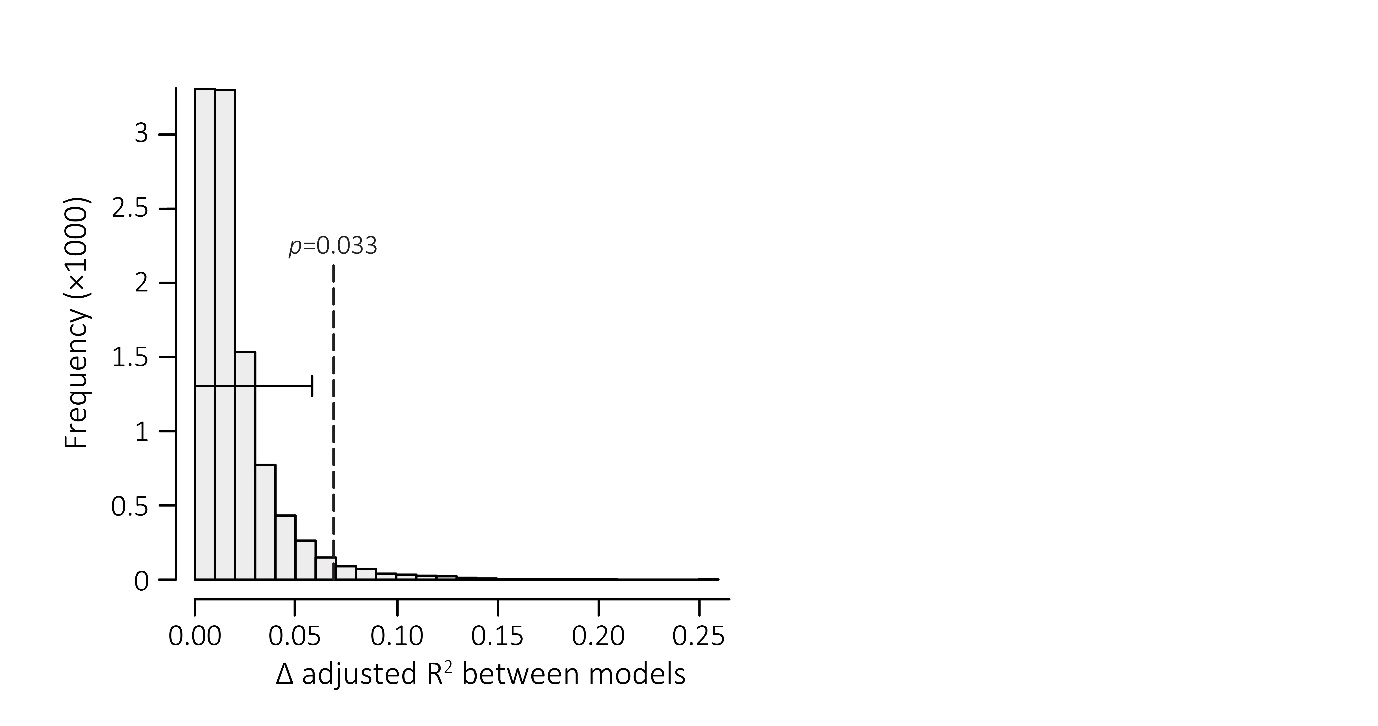
**

Histogram of permuted absolute (i.e., |x|) differences in adjusted explained variance (R^2^) between the two selected best models (i.e., ΔPVR interaction model versus baseline PVR main effect model). The distribution reflects expected differences in R^2^ between models, under the null hypothesis (H0; no link between predictors and outcome). The error bar indicates the 95% confidence interval of the null distribution. The dashed line marks the observed difference in R^2^.

**Supplementary Tables S1-S12. Overview of model performance and predictor contributions**

*Baseline PVR main effect (baselinePVR + age + sex)*

Table S1 – Model fit statistics:

| Model | R² | Adj. R² | AIC | BIC | *p*-value |
| --- | --- | --- | --- | --- | --- |
| Baseline PVR main effect | 0.133 | 0.091 | 148.1 | 159.0 | 0.030 |

Table S2 – Coefficient estimates and predictive strength:

|  |  |  | 95% conf. int. | |  |  |  | Bootstrap | | | |
| --- | --- | --- | --- | --- | --- | --- | --- | --- | --- | --- | --- |
| Parameter | Estimate (β) | Std Error | Lower | Upper | *p*-value | Std Estimate (β) | Partial R² | Median Partial R² | Std Error Bootstrap R² | Lower IQR R² | Upper IQR R² |
| (Intercept) | 0.046 | 0.524 | -1.001 | 1.093 | 0.930 | - | - | - | - | - | - |
| baselinePVR | -0.043 | 0.017 | -0.077 | -0.010 | 0.013 | -0.311 | 0.096 | 0.098 | 0.065 | 0.059 | 0.147 |
| age | 0.003 | 0.006 | -0.009 | 0.016 | 0.597 | 0.064 | - | - | - | - | - |
| sex | -0.335 | 0.210 | -0.754 | 0.084 | 0.115 | -0.190 | - | - | - | - | - |

*Baseline PVR interaction (baselinePVR×age + baselinePVR + age + sex)*

Table S3 – Model fit statistics:

| Model | R² | Adj. R² | AIC | BIC | *p*-value |
| --- | --- | --- | --- | --- | --- |
| Baseline PVR interaction | 0.140 | 0.083 | 149.6 | 162.7 | 0.053 |

Table S4 – Coefficient estimates and predictive strength:

|  |  |  | 95% conf. int. | |  |  |  | Bootstrap | | | |
| --- | --- | --- | --- | --- | --- | --- | --- | --- | --- | --- | --- |
| Parameter | Estimate (β) | Std Error | Lower | Upper | *p*-value | Std Estimate (β) | Partial R² | Median Partial R² | Std Error Bootstrap R² | Lower IQR R² | Upper IQR R² |
| (Intercept) | 0.555 | 0.909 | -1.262 | 2.373 | 0.543 | - | - | - | - | - | - |
| baselinePVR | -0.084 | 0.062 | -0.207 | 0.039 | 0.178 | -0.602 | 0.030 | 0.030 | 0.068 | 0.007 | 0.083 |
| baselinePVR×age | 0.001 | 0.001 | -0.001 | 0.003 | 0.495 | 0.316 | 0.008 | 0.017 | 0.048 | 0.004 | 0.052 |
| age | -0.005 | 0.014 | -0.032 | 0.022 | 0.712 | -0.098 | - | - | - | - | - |
| sex | -0.344 | 0.211 | -0.766 | 0.078 | 0.108 | -0.195 | - | - | - | - | - |

*4-month PVR main effect (4-monthPVR + age + sex)*

Table S5 – Model fit statistics:

| Model | R² | Adj. R² | AIC | BIC | *p*-value |
| --- | --- | --- | --- | --- | --- |
| 4-month PVR main effect | 0.057 | 0.011 | 153.7 | 164.6 | 0.303 |

Table S6 – Coefficient estimates and predictive strength:

|  |  |  | 95% conf. int. | |  |  |  | Bootstrap | | | |
| --- | --- | --- | --- | --- | --- | --- | --- | --- | --- | --- | --- |
| Parameter | Estimate (β) | Std Error | Lower | Upper | *p*-value | Std Estimate (β) | Partial R² | Median Partial R² | Std Error Bootstrap R² | Lower IQR R² | Upper IQR R² |
| (Intercept) | -0.293 | 0.570 | -1.433 | 0.846 | 0.609 | - | - | - | - | - | - |
| 4-monthPVR | -0.033 | 0.033 | -0.098 | 0.032 | 0.312 | -0.130 | 0.016 | 0.025 | 0.059 | 0.005 | 0.077 |
| age | 0.005 | 0.006 | -0.008 | 0.018 | 0.449 | 0.096 | - | - | - | - | - |
| sex | -0.326 | 0.221 | -0.767 | 0.116 | 0.145 | -0.185 | - | - | - | - | - |

*4-month PVR interaction (4-monthPVR×age + 4-monthPVR + age + sex)*

Table S7 – Model fit statistics:

| Model | R² | Adj. R² | AIC | BIC | *p*-value |
| --- | --- | --- | --- | --- | --- |
| 4-month PVR interaction | 0.098 | 0.039 | 152.7 | 165.8 | 0.171 |

Table S8 – Coefficient estimates and predictive strength:

|  |  |  | 95% conf. int. | |  |  |  | Bootstrap | | | |
| --- | --- | --- | --- | --- | --- | --- | --- | --- | --- | --- | --- |
| Parameter | Estimate (β) | Std Error | Lower | Upper | *p*-value | Std Estimate (β) | Partial R² | Median Partial R² | Std Error Bootstrap R² | Lower IQR R² | Upper IQR R² |
| (Intercept) | -1.076 | 0.730 | -2.537 | 0.384 | 0.146 | - | - | - | - | - | - |
| 4-monthPVR | 0.077 | 0.073 | -0.069 | 0.223 | 0.297 | 0.301 | 0.018 | 0.045 | 0.056 | 0.021 | 0.086 |
| 4-monthPVR×age | -0.002 | 0.001 | -0.005 | 0.000 | 0.099 | -0.512 | 0.044 | 0.059 | 0.053 | 0.029 | 0.097 |
| age | 0.020 | 0.011 | -0.002 | 0.042 | 0.074 | 0.391 | - | - | - | - | - |
| sex | -0.331 | 0.218 | -0.766 | 0.105 | 0.134 | -0.187 | - | - | - | - | - |

*ΔPVR main effect (ΔPVR + age + sex)*

Table S9 – Model fit statistics:

| Model | R² | Adj. R² | AIC | BIC | *p*-value |
| --- | --- | --- | --- | --- | --- |
| ΔPVR main effect | 0.134 | 0.093 | 148.0 | 158.9 | 0.029 |

Table S10 – Coefficient estimates and predictive strength:

|  |  |  | 95% conf. int. | |  |  |  | Bootstrap | | | |
| --- | --- | --- | --- | --- | --- | --- | --- | --- | --- | --- | --- |
| Parameter | Estimate (β) | Std Error | Lower | Upper | *p*-value | Std Estimate (β) | Partial R² | Median Partial R² | Std Error Bootstrap R² | Lower IQR R² | Upper IQR R² |
| (Intercept) | -0.295 | 0.472 | -1.239 | 0.649 | 0.534 | - | - | - | - | - | - |
| ΔPVR | 0.057 | 0.022 | 0.013 | 0.100 | 0.012 | 0.308 | 0.098 | 0.097 | 0.072 | 0.053 | 0.151 |
| age | 0.005 | 0.006 | -0.007 | 0.017 | 0.447 | 0.091 | - | - | - | - | - |
| sex | -0.287 | 0.209 | -0.704 | 0.130 | 0.174 | -0.162 | - | - | - | - | - |

*ΔPVR interaction (ΔPVR×age + ΔPVR + age + sex)*

Table S11 – Model fit statistics:

| Model | R² | Adj. R² | AIC | BIC | *p*-value |
| --- | --- | --- | --- | --- | --- |
| ΔPVR interaction | 0.212 | 0.160 | 143.8 | 157.0 | 0.005 |

Table S12 – Coefficient estimates and predictive strength:

|  |  |  | 95% conf. int. | |  |  |  | Bootstrap | | | |
| --- | --- | --- | --- | --- | --- | --- | --- | --- | --- | --- | --- |
| Parameter | Estimate (β) | Std Error | Lower | Upper | *p*-value | Std Estimate (β) | Partial R^2^ | Median Partial R² | Std Error Bootstrap R² | Lower IQR R² | Upper IQR R² |
| (Intercept) | 0.689 | 0.607 | -0.525 | 1.904 | 0.261 | - | - | - | - | - | - |
| ΔPVR | 0.253 | 0.083 | 0.087 | 0.419 | 0.003 | 1.374 | 0.132 | 0.118 | 0.094 | 0.050 | 0.195 |
| ΔPVR×age | -0.003 | 0.001 | -0.006 | -0.001 | 0.017 | -1.117 | 0.089 | 0.078 | 0.076 | 0.028 | 0.141 |
| age | -0.012 | 0.009 | -0.029 | 0.006 | 0.195 | -0.227 | - | - | - | - | - |
| sex | -0.303 | 0.201 | -0.705 | 0.099 | 0.137 | -0.172 | - | - | - | - | - |
